# Practical Points

**Published:** 1907-10-19

**Authors:** 


### PRACTICAL POINTS.

### HOW TO COMBAT THE AFTER-LOCAL EFFECT OF COCAINE.

By Fred. Page Atkinson, M.D., M.R.C.P.Edin., Bexhill-on-Sea.

One great drawback to the use of cocaine in nasal operations is the swelling of the mucous membrane which afterwards occurs, and I have questioned several medical men as to what they do in these cases, but have not so far met with any satisfactory response. However, after a series of trials I have come to the conclusion that the best way of overcoming the worry and annoyance is to administer from 10 to 15 grains of veronal within four hours after the operation, which is generally the time at which the swelling begins to take place. This not only gives a night's rest, but also has a soothing local effect, no doubt through nerve influence, the next day.

